# Quorum Sensing, Virulence, and Antibiotic Resistance of USA100 Methicillin-Resistant Staphylococcus aureus Isolates

**DOI:** 10.1128/mSphere.00553-19

**Published:** 2019-08-14

**Authors:** Morgan L. Grundstad, Corey P. Parlet, Jakub M. Kwiecinski, Jeffrey S. Kavanaugh, Heidi A. Crosby, Young-Saeng Cho, Kristopher Heilmann, Daniel J. Diekema, Alexander R. Horswill

**Affiliations:** a University of Iowa Stead Family Children’s Hospital, Iowa City, Iowa, USA; b Department of Microbiology and Immunology, University of Iowa, Iowa City, Iowa, USA; c Department of Immunology and Microbiology, University of Colorado School of Medicine, Aurora, Colorado, USA; d Department of Veterans Affairs Eastern Colorado Healthcare System, Aurora, Colorado, USA; e Division of Infectious Diseases, Department of Internal Medicine, University of Iowa Carver College of Medicine, Iowa City, Iowa, USA; f Division of Medical Microbiology, Department of Pathology, University of Iowa Carver College of Medicine, Iowa City, Iowa, USA; University of Rochester

**Keywords:** MRSA, USA100, *agr*, quorum sensing

## Abstract

Methicillin-resistant Staphylococcus aureus (MRSA) infections impact all patient populations both in the community and in health care settings. Despite advances in our knowledge of MRSA virulence, little is known about the regulatory mechanisms of USA100 health care-associated MRSA isolates, which are the second most frequently identified MRSA isolates found in all infections. This work focused on the contribution of the USA100 *agr* type II quorum-sensing system to virulence and antibiotic resistance. From a MRSA strain collection, we selected 16 representative USA100 isolates, constructed mutants with Δ*agr* mutations, and characterized selected strain pairs for virulence factor expression, murine skin infection, and antibiotic resistance. For each strain pair, hemolysis and extracellular protease expression were significantly greater in the wild-type (WT) strains than in the Δ*agr* mutants. Similarly, mice challenged with the WT strains had larger areas of dermonecrosis and greater weight loss than those challenged with the Δ*agr* mutants, demonstrating that the USA100 *agr* system regulates virulence. Although USA100 isolates exhibit a high level of antibiotic resistance, the WT and Δ*agr* strain pairs showed no difference in MICs by MIC testing. However, in the presence of a sub-MIC of vancomycin, most of the USA100 Δ*agr* mutants exhibited slower growth than the WT isolates, and a couple of the Δ*agr* mutants also grew more slowly in the presence of a sub-MIC of cefoxitin. Altogether, our findings demonstrate that the USA100 *agr* system is a critical regulator of virulence, and it may have a contribution to the optimal survival of these MRSA strains in the presence of antibiotics.

**IMPORTANCE** USA100 health care-associated MRSA isolates are highly antibiotic resistant and can cause invasive disease across all patient populations. Even though USA100 strains are some of the most frequently identified causes of infections, little is known about virulence regulation in these isolates. Our study demonstrates that the USA100 *agr* quorum-sensing system is important for the control of toxin and exoenzyme production and that the *agr* system has a key role in skin infection. In some USA100 isolates, the *agr* system is important for growth in the presence of low levels of antibiotics. Altogether, our findings demonstrate that the USA100 *agr* system is a critical regulator of virulence and that it may make a contribution to the optimal survival of these MRSA strains in the presence of antibiotics.

## Abstract (precis)

USA100 health care-associated MRSA isolates are highly antibiotic resistant and can cause invasive disease across all patient populations. Even though USA100 strains are some of the most frequently identified causes of infections, little is known about virulence regulation in these isolates. Our study demonstrates that the USA100 *agr* quorum-sensing system is important for the control of toxin and exoenzyme production and that the *agr* system has a key role in skin infection. In some USA100 isolates, the *agr* system is important for growth in the presence of low levels of antibiotics. Altogether, our findings demonstrate that the USA100 *agr* system is a critical regulator of virulence and that it may make a contribution to the optimal survival of these MRSA strains in the presence of antibiotics.

## INTRODUCTION

Staphylococcus aureus is a Gram-positive opportunistic bacterial pathogen known to cause a diverse array of acute and chronic infections (1, 2). Exposure to antibiotics has resulted in the evolution of strains, producing strains such as methicillin-resistant Staphylococcus aureus (MRSA), which has become the cause of a global health care epidemic (3, 4). Over the past 2 decades, these MRSA strains have moved into the community and have begun infecting otherwise healthy individuals, resulting in a new wave of resistant strains called community-associated MRSA (CA-MRSA). CA-MRSA strains, the most important of which are the USA300 group of isolates, have spread globally and are responsible for severe and devastating disease (1, 3–5).

Due to the attention garnered by the emergence of CA-MRSA and the subsequent spread of common CA-MRSA strains (e.g., USA300 isolates) into health care settings, recent research has focused on understanding the virulence mechanisms enabling these isolates to be so successful. Meanwhile, strain types that more commonly cause health care-associated MRSA (HA-MRSA) infections, which have been around much longer and which have remained more confined to hospital settings, have not received as much basic research attention, despite the frequency with which they are responsible for infections. The most common HA-MRSA strains are those of the USA100 pulsed-field gel type, which have a staphylococcal cassette chromosome *mec* (SCC*mec*) type II island, are *spa* type 2, and are often multidrug resistant (6, 7). In 2005 and 2006, 53.2% of the MRSA samples from patients with invasive disease were of the USA100 genotype (8). In 2009 and 2010, 37% of nasal MRSA isolates and 36% of blood culture MRSA isolates from one study were USA100 (7). In a related study, Richter et al. found that USA100 isolates represented 16.8% of MRSA isolates and were the most common blood culture MRSA isolates (9). In a more recent comprehensive meta-analysis, USA100 MRSA levels were found to have remained fairly steady from 2005 to 2013, ranging from 21 to 25% of MRSA collections, second only to the USA300 isolates in each time frame (10).

In comparison to USA300 isolates, the USA100 isolates demonstrate *in vitro* resistance to more antibiotic classes, such as fluoroquinolones, macrolides, and lincosamides (9). Additionally, most S. aureus isolates that demonstrate vancomycin resistance or intermediate resistance are of the USA100 genotype (11). However, in terms of infection potential, HA-MRSA isolates and representative USA100 isolates are generally considered less virulent and secrete cytotoxins at lower levels than their CA-MRSA counterparts (12, 13), characteristics which have resulted in limited interest in the HA-MRSA virulence mechanisms.

**FIG 1:**
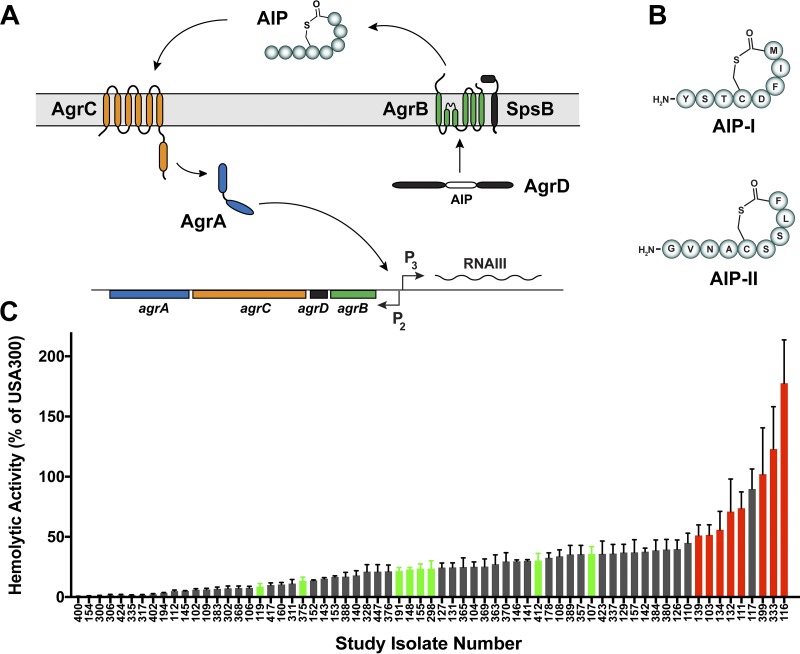
The USA100 *agr* type II system. (A) Schematic of the S. aureus *agr* system. (B) Structures of the AIP-I signal from USA300 strains and the AIP-II signal from USA100 strains. (C) Alpha-toxin levels from a rabbit red blood cell hemolysis assay of the 67 strains that met the inclusion criteria. The data are plotted as a percentage of activity relative to that for USA300 strain LAC, which was set to 100%. The 16 USA100 strains for which results are presented in red and green were selected for further study.

In all S. aureus strains, toxin regulation is controlled by a peptide quorum-sensing system (Fig. 1A), also called the accessory gene regulator (*agr*) (14–16). The system responds to a secreted autoinducing peptide (AIP) signal generated from the AgrD peptide precursor by the AgrB membrane endopeptidase. At a critical concentration, the AIP binds to a receptor on the AgrC histidine kinase, activating the kinase, which in turn phosphorylates the response regulator AgrA. Activated AgrA in turn binds the P2 and P3 promoters in the *agr* locus, autoinducing the system and also upregulating the RNAIII effector, which turns on toxin and exoenzyme production. Across S. aureus strains, there are hypervariable regions that exist within the *agrB*, *agrD*, and *agrC* genes, and this results in four different locus variants, called *agr* type I (*agr*-I), *agr*-II, *agr*-III, and *agr*-IV. In turn, these loci produce four different AIP types, called AIP-I through AIP-IV (17). The common CA-MRSA isolate USA300 has the *agr*-I allele and produces AIP-I, while USA100 isolates utilize the *agr*-II system and produce AIP-II (Fig. 1B). Interestingly, the type I and II systems remain the most divergent across S. aureus strains in terms of sequence (14).

Our goal in this study was to better understand the contribution of the *agr*-II system to USA100 MRSA antibiotic resistance and virulence. We obtained clinical USA100 isolates that represent *spa* type 2 and SCC*mec* type II and selected those that had a functional *agr* system, as predicted from toxin production. There is some evidence that *agr* contributes to antibiotic resistance (18), and since USA100 isolates are some of the most resistant isolates, we reasoned that *agr*-II might influence resistance levels. Since *agr* is a major contributor to the virulence of other MRSA isolates, we predicted that it would be important for toxin and exoenzyme production, as well as infection potential.

## RESULTS

### Bacterial strain selection and construction of *agr* mutants.

A total of 526 pulsed-field gel electrophoresis- and *spa*-typed S. aureus strains were provided by the University of Iowa Molecular Epidemiology Laboratory (19–21). Out of this collection, 146 were *spa* type 2, representing USA100 isolates. The collection was comprised of roughly equal numbers of colonizing and invasive isolates, and only the invasive ones (blood culture isolates) were considered further. Of these, only USA100 strains that contained SCC*mec* type II (MRSA) and that were tetracycline sensitive (for *agr* mutant construction) were selected for further testing, eliminating many isolates and narrowing the collection to 67 candidates.

HA-MRSA strains are known to pick up *agr* locus variants that result in dysfunction more commonly than CA-MRSA strains (22, 23). Since the goal was to study USA100 isolates with an intact *agr* system, we assessed the alpha-toxin activity of the 67 candidates as a proxy for *agr* function. The results of a rabbit red blood cell lysis assay serve as an indicator of alpha-toxin activity (24), and we performed this assay with the candidates and compared the results to those for USA300 strain LAC as a control (Fig. 1C). Eight USA100 isolates that had robust alpha-toxin production (marked in red in Fig. 1C; Table 1), suggesting that they had intact and functional *agr* type II systems, were selected. To inactivate the *agr* locus, the well-known Δ*agr*::TetM (hereafter called Δ*agr*) cassette was crossed into each strain, and the mutation was confirmed. An additional eight strains that had lower levels of alpha-toxin production (marked in green in Fig. 1C; Table 1) were selected for comparison. Table 2 summarizes the constructed USA100 wild-type (WT) and *agr* null strain pairs.

**TABLE 1:** Characteristics of USA100 isolates selected for study^*a*^

Study clinical isolate no.	Source	*spa* type	SCC*mec* type	PVL	% of LAC RBC lysis activity
111	Blood	t002	II	Negative	73.8
103	Blood	t002	II	Negative	51.5
116	Blood	t002	II	Negative	177.6
132	Blood	t002	II	Negative	70.9
134	Blood	t002	II	Negative	55.9
139	Blood	t002	II	Negative	51.1
333	Blood	t002	II	Positive	123
399	Blood	t002	II	Positive	102
119	Blood	t002	II	Negative	8.7
375	Blood	t002	II	Positive	13.5
191	Blood	t088	II	Negative	21.5
148	Blood	t002	II	Negative	22.9
155	Blood	t002	II	Negative	23.6
298	Blood	t002	II	Positive	23.6
412	Blood	t002	II	Positive	30.3
107	Blood	t002	II	Negative	35.7

a PVL, Panton-Valentine leukocidin; RBC, red blood cell.

**TABLE 2:** Nomenclature for the USA100 wild-type and *agr* mutant isolates studied

Strain	Study clinical isolate no.	Reference or source
AH1263	LAC (Erm sensitive)^*a*^	51
AH843	MW2	52
AH2759	LAC/pAmiAgrP3	53
AH1677	LAC/pDB59	27
AH3185	MW2/pAmiAgrP3	This work
AH1747	MW2/pDB59	27
AH4489	103	This work
AH4541	103 Δ*agr*	This work
AH5445	103/pDB59	This work
AH2617	111	This work
AH2618	111 Δ*agr*	This work
AH5510	111/pDB59	This work
AH4490	116	31
AH4542	116 Δ*agr*	This work
AH5446	116/pDB59	This work
AH4390	116/pAmiAgrP3	This work
AH4491	132	31
AH4543	132 Δ*agr*	This work
AH5492	132/pDB59	This work
AH4492	134	This work
AH4544	134 Δ*agr*	This work
AH5447	134/pDB59	This work
AH5070	134/pAmiAgrP3	This work
AH4493	139	This work
AH4528	139 Δ*agr*	This work
AH5448	139/pDB59	This work
AH4494	333	This work
AH4529	333 Δ*agr*	This work
AH5493	333/pDB59	This work
AH4495	399	This work
AH4530	399 Δ*agr*	This work
AH5299	412	This work
AH5495	412/pDB59	This work
AH5294	155	This work
AH5494	155/pDB59	This work
AH5300	107	This work
AH5453	107/pDB59	This work
AH5298	375	This work
AH5452	375/pDB59	This work
AH5296	298	This work
AH5451	298/pDB59	This work
AH5295	191	This work
AH5450	191/pDB59	This work
AH5297	119	This work
AH5293	148	This work

a Erm, erythromycin.

### USA100 *agr* system kinetics.

**FIG 2:**
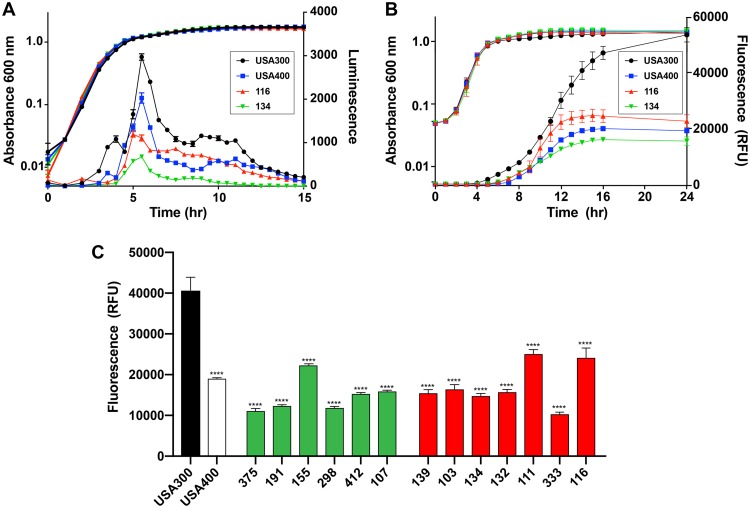
USA100 *agr* activation kinetics. (A) USA300 strain LAC, USA400 strain MW2, and USA100 strains 116 and 134 containing an *agr* P3-*lux* reporter were grown for 15 h, and the optical density at 600 nm and luminescence were measured. (B) USA300 strain LAC, USA400 strain MW2, and USA100 strains 116 and 134 containing the *agr* P3-YFP reporter (pDB59) were grown for 24 h, and the optical density at 600 nm and the fluorescence were measured. (C) Fluorescence measurements of all USA100 strains used in the study containing the *agr* P3-YFP reporter (pDB59) after 14 h of growth. Statistical significance was determined using Student's *t* test. ****, *P* < 0.0001. RFU, relative fluorescence units.

To compare the *agr* activation kinetics in USA100 isolates to that in other S. aureus strains, we moved *agr* P3-*lux* reporter constructs into selected USA100 strains (strains 116 and 134), USA300 LAC, and USA400 MW2. As shown in Fig. 2A, each *agr* P3-*lux* reporter strain activated the *agr* system in the exponential phase of growth, and maximal activity was reached at about 5 to 6 h.

While the *agr* P3-*lux* reporter is powerful and provides useful kinetic analysis, it is somewhat challenging to use genetically, as some S. aureus strains do not take up the plasmid. To have a broader comparison, we used the pDB59 *agr* P3-yellow fluorescent protein (YFP) reporter, which has been our workhorse reporter for quorum-sensing assessments (25–27). We moved the pDB59 plasmid into all 16 USA100 strains (8 high-toxin producers and 8 low- to middle-level toxin producers; Tables 1 and 2). Strains 119 and 148 had no *agr* activity, which we confirmed with secondary methods, and strain 399 grew poorly with the pDB59 plasmid and was not tested further. Comparison of strains 116 and 134 with *agr* P3-*lux* (Fig. 2A) with strains 116 and 134 with *agr* P3-YFP (Fig. 2B) revealed delayed activation with the slowly folding YFP, with *lux* coming on at about 4 h, while YFP came on at about 10 h. The USA300 strains had the strongest *agr* output with both reporters, while either the USA400 or the USA100 116 strain (the highest toxin producer) had the next-strongest output (Fig. 2A and B), and the USA100 134 strain had the weakest output of this set.

Next, we compared the 13 USA100 YFP reporter strains to the CA-MRSA controls, selecting the 14-h time point to assess the maximal activity of the reporter (Fig. 2C). The activity of the USA300 reporter was consistently and significantly higher than that of all others at this time point. Some of the high-alpha-toxin-producing USA100 isolates (strains 111 and 116) maintained a high *agr* output, while some of the low-toxin producers (strains 191 and 375) had a low *agr* output or lacked *agr* function (strains 119 and 148). However, there was a lot of strain-dependent variability that was not always consistent (Fig. 2C). In general, the CA-MRSA strains are known to have a robust *agr* system function and to produce high levels of RNAIII (14), and within the CA-MRSA group, the USA300 lineage has an exceptionally strong *agr* function that can outperform that of isolates of other lineages, such as USA400 isolates (28). Taken together, USA100 isolates have *agr* kinetics of activation similar to that of CA-MSRA isolates but achieve a lower overall maximal output.

### USA100 virulence factor production.

**FIG 3:**
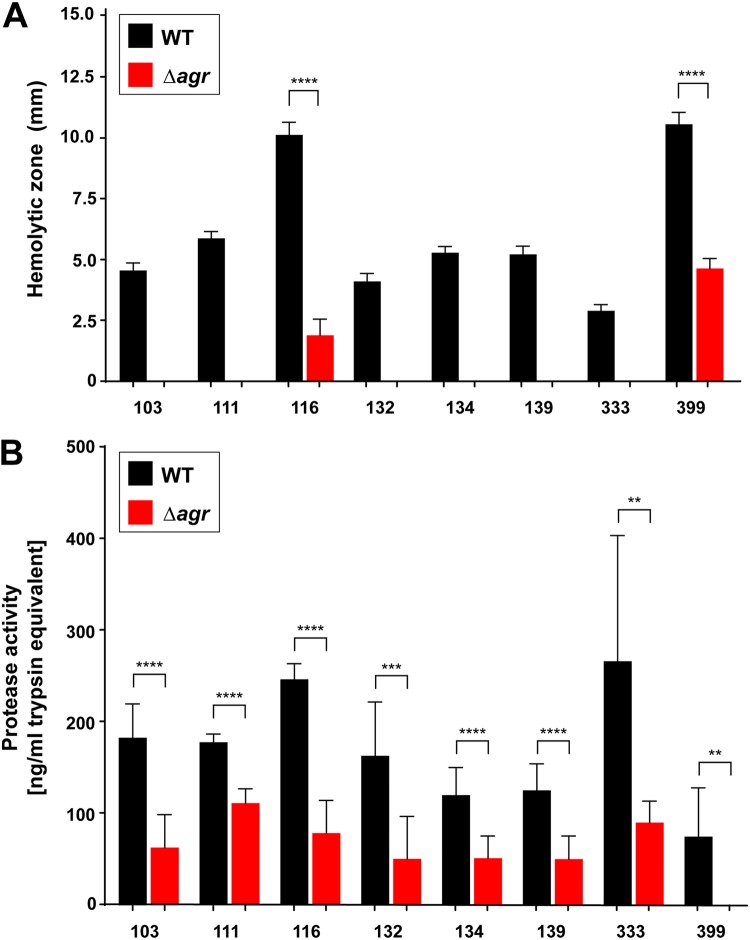
Contribution of *agr* to USA100 virulence factor expression. (A) Hemolytic zones at 24 h, measured as the diameter (in millimeters) minus the colony size (in millimeters) for each pair. (B) Proteolytic activity, expressed as the equivalent of a trypsin standard. The WT demonstrated more hemolysis and proteolysis than the Δ*agr* mutant in the strain pairs, but two Δ*agr* mutants (strains 116 and 399) did show low levels of hemolysis. Statistical significance was determined using Student's *t* test. ****, *P* < 0.0001; ***, *P < *0.001; **, *P < *0.01.

It is known that the *agr* system regulates hemolysins and exoenzymes that are essential for S. aureus virulence (14). The suite of *agr*-regulated hemolysins is diverse and includes alpha-toxin, β-hemolysin, gamma-hemolysin, phenol-soluble modulins (PSMs), and the other bicomponent leukocidins. As reported previously (29, 30), we used a qualitative sheep blood hemolysis assay to broadly assess the *agr* contribution to USA100 hemolysin production. For each USA100 WT and Δ*agr* strain pair, clearing zones were measured at 24 and 48 h, and as anticipated, the WT strains demonstrated significantly more hemolysis than the *agr* mutant strains (Fig. 3A). Interestingly, two *agr* mutants, 116 Δ*agr* and 399 Δ*agr*, had background hemolysis at 24 and 48 h, although it was significantly less than that of the WT. None of the other Δ*agr* mutants had background hemolysis. We reasoned that some of the hemolysis occurring in the Δ*agr* mutants might be due to β-hemolysin, which is not as tightly *agr* regulated (30). The *hlb* gene, encoding β-hemolysin, is often disrupted in clinical isolates by an integrated bacteriophage, such as the ϕSa3-related phages (31, 32), and these phages encode additional virulence factors, such as staphylokinase (SAK) (32). To determine the presence of ϕSa3-related phages in the USA100 strains (high alpha-toxin producing), PCR was performed on all the USA100 WT strains, which confirmed the presence of the *sak* gene in all strains except the 399 strain. Furthermore, the *hlb* gene was successfully identified to be intact in the 399 WT strain.

Secreted enzymes are another major class of S. aureus virulence factors regulated by the *agr* system (33, 34). We focused on the extracellular proteases, and we used a fluorescent casein cleavage assay to assess activity in the USA100 strain pairs. As shown in Fig. 3B, the USA100 WT strains had more extracellular protease activity than the Δ*agr* mutant strains, although, surprisingly, many of the Δ*agr* mutants had substantial background activity. It should be noted, though, that not every protease secreted by S. aureus cleaves casein with a similar efficiency. Based on the results of these different assays, overall, the USA100 *agr* type II system behaves similarly to that of other S. aureus strain lineages in terms of virulence factor expression.

### The USA100 *agr* system contributes to skin infection.

**FIG 4:**
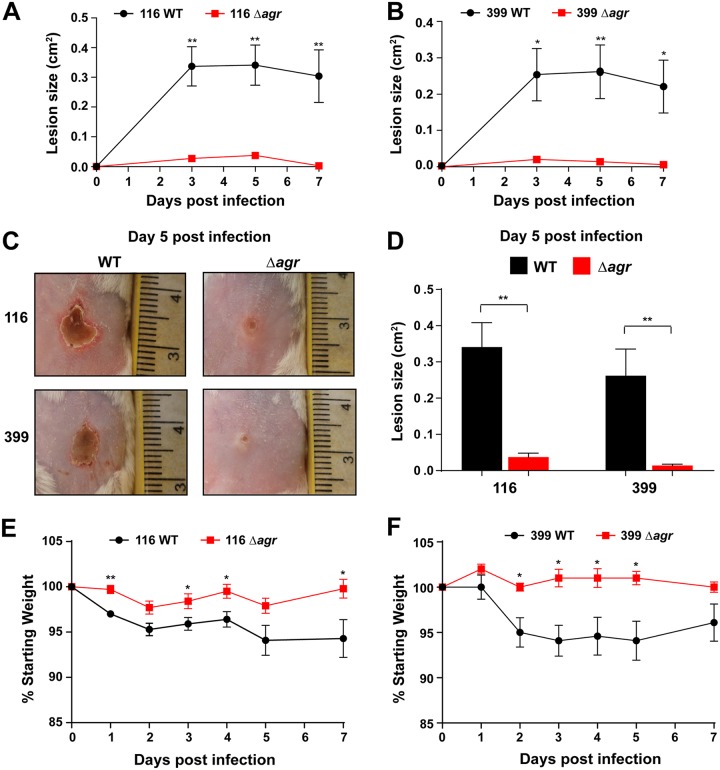
Role of *agr* in USA100 skin infections. (A, B) Comparison of lesion size for mice inoculated with the strain 116 (A) and 399 (B) WT and Δ*agr* mutant strain pairs over 1 week (*n* = 5 for each group). (C) Photographs of ventral skin lesions 5 days after inoculation with WT and Δ*agr* mutant strains. Note the smaller areas of dermal necrosis resulting from infection with the Δ*agr* mutants. (D) Comparison of lesion size between WT and Δ*agr* mutant isolates 5 days after intradermal injection of mice (*n* = 5 for each group). (E, F) Comparison of weight loss (expressed as a percentage of the starting weight) in mice which underwent intradermal injections of the strain 116 (E) and 399 (F) WT and Δ*agr* isolates. Statistical significance was determined using Student's *t* test. **, *P < *0.01; *, *P < *0.05.

Numerous studies have demonstrated that the *agr* system of CA-MRSA USA300 strains contributes to skin infection in animal models (35–39), but its contribution is less clear in the HA-MRSA USA100 isolates. Specifically, we chose the strain pairs of the two strains 116 and 399 as USA100 strain sets that exhibited a high level of hemolysis both for the WT and at the background level for the Δ*agr* mutant (Fig. 3A). Using an intradermal infection model that we have previously used to assess MRSA skin virulence (36–38, 40, 41), the lesion sizes were dramatically bigger in mice challenged with the 116 WT strain than in mice challenged with the Δ*agr* mutant over time (Fig. 4A). A similar pattern was observed with the 399 strain, with the lesions in the mice challenged with the WT being consistently larger than those in the mice challenged with the Δ*agr* mutant (Fig. 4B). Pictures of the gross lesion pathology (Fig. 4C) at 5 days and measurement of these lesion sizes (Fig. 4D) confirmed this phenotype. Consistent with this, the USA100 WT-challenged mice experienced more weight loss over 7 days than the mutant strain-challenged mice (Fig. 4E and F). Despite each of these strains having significant background levels of hemolysis on sheep blood (Fig. 3A), the contribution of this to a skin lesion was minimal. Taken together, these data suggest that when the *agr* system is intact, higher levels of virulence factors are expressed, resulting in a greater systemic effect of infection, in addition to a larger abscess size.

### Contribution of *agr* to antibiotic resistance.

Previous studies have suggested a potential difference in the survival of MRSA WT strains and Δ*agr* strains when exposed to antibiotics (18, 23, 42). As shown in Table 3, we tested the USA100 strain pairs for resistance to 13 common antibiotics using Etest strips. Except for the tetracycline resistance due to the Δ*agr*::TetM construct, there was no significant difference in antibiotic resistance between the USA100 WT and each paired Δ*agr* mutant. This matches previous reports of the results of MIC testing of diverse S. aureus strains and Δ*agr* mutants (42).

**TABLE 3:** MICs of USA100 strain pairs

Strain	MIC^*a*^ (μg/ml)												
	FOX	CLI	DAP	ERY	GEN	LVX	LZD	OXA	Q-D	RIF	TET	SXT	VAN
103 WT	96	0.094	0.25	>256	0.25	>32	1.5	>256	0.5	0.012	0.19	0.064	1.5
103 Δ*agr*	48	0.094	0.125	>256	0.25	>32	2	>256	0.5	0.012	24	0.064	1
111 WT	12	> 256	0.38	>256	0.38	>32	3	>256	1	0.016	0.5	0.094	2
111 Δ*agr*	32	>256	0.19	>256	0.5	>32	3	192	1	0.012	32	0.064	1.5
116 WT	48	0.125	0.19	>256	0.38	>32	1.5	64	0.38	0.008	0.25	0.094	1
116 Δ*agr*	24	0.094	0.125	>256	0.5	>32	1.5	24	0.38	0.008	24	0.094	1
132 WT	>256	0.125	0.19	>256	0.38	>32	2	>256	1.5	0.012	0.38	0.094	1.5
132 Δ*agr*	>256	0.125	0.25	>256	0.75	>32	2	>256	0.75	0.012	48	0.094	1.5
134 WT	>256	0.125	0.25	>256	0.38	>32	2	>256	0.5	0.012	0.19	0.094	2
134 Δ*agr*	>256	0.125	0.25	>256	0.38	>32	1.5	>256	0.5	0.012	24	0.094	2
139 WT	>256	>256	0.38	>256	0.38	>32	3	>256	1	0.012	0.38	0.094	2
139 Δ*agr*	>256	>256	0.38	>256	0.5	>32	3	>256	0.75	0.012	24	0.094	1.5
333 WT	192	0.125	0.38	256	0.5	>32	2	>256	0.5	0.012	0.5	0.094	2
333 Δ*agr*	96	0.125	0.38	>256	0.38	>32	2	>256	0.5	0.012	16	0.094	1.5
399 WT	>256	>256	0.5	>256	0.75	8	1.5	>256	0.75	0.012	0.19	0.19	2
399 Δ*agr*	>256	>256	0.38	>256	0.75	6	1.5	>256	0.75	0.012	24	0.19	2

a MICs are based on the Etest for the USA100 wild-type and *agr* mutant isolate pairs. Overall, there was no difference in antibiotic resistance, as determined by the use of Etest strips, with the exception of resistance to tetracycline, which was as expected, since the tetracycline resistance cassette was incorporated into the genome of each *agr* mutant as part of mutant construction. FOX, cefoxitin; CLI, clindamycin; DAP, daptomycin; ERY, erythromycin; GEN, gentamicin; LVX, levofloxacin; LZD, linezolid; OXA, oxacillin; Q-D, quinupristin-dalfopristin; RIF, rifampin; TET, tetracycline; SXT, trimethoprim-sulfamethoxazole; VAN, vancomycin.

**FIG 5:**
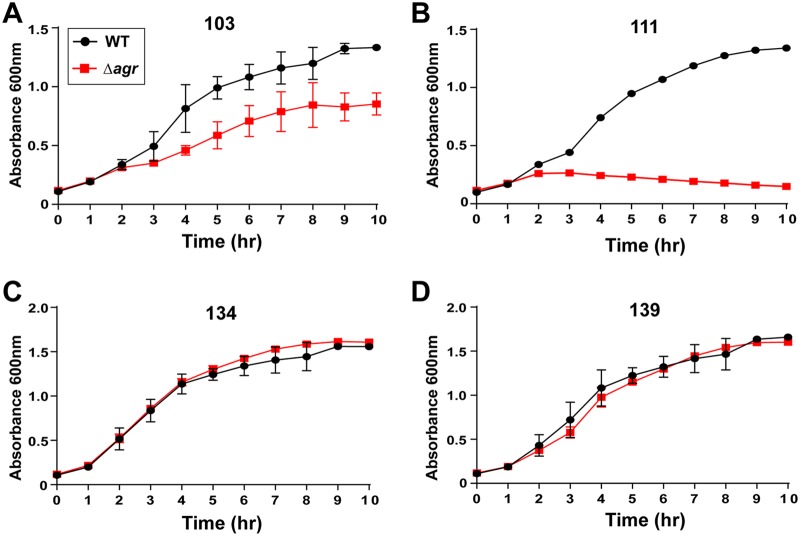
Growth of USA100 strains in the presence of a sub-MIC of cefoxitin. (A, B) The strain 103 (A) and 111 (B) WT strains demonstrated growth superior to that of the paired Δ*agr* mutants when exposed to cefoxitin, suggesting that a functional *agr* system in these strains contributes to survival under antibiotic stress. (C, D) Growth curves for representative strains 134 (C) and 139 (D) in which no differences in growth were observed between each of the strains in the strain pairs tested with the same concentration of cefoxitin.

Since survival and stress assays have shown different behaviors for strains with a defective *agr* gene, we performed more careful growth curves for each USA100 strain pair with cefoxitin, clindamycin, daptomycin, and vancomycin. For cefoxitin at levels below the MIC (∼16 μg/ml), some differences in growth were noted. The Δ*agr* mutant and the WT in the strain 103 strain pair demonstrated statistically significantly different inhibitory growth (Fig. 5A), and the two strains in the strain 333 strain pair grew in a fashion comparable to that for the two strains in the strain 103 strain pair (data not shown). For the strain 111 strain pair, a similar and even more pronounced pattern was apparent, with the growth of the Δ*agr* mutant dramatically lagging behind that of the WT (Fig. 5B). The growth of the WT and the Δ*agr* mutant in the rest of the strain pairs exhibited few differences in the presence of cefoxitin (representative plots for strains 134 and 139 are shown in Fig. 5C and D, respectively.

**FIG 6:**
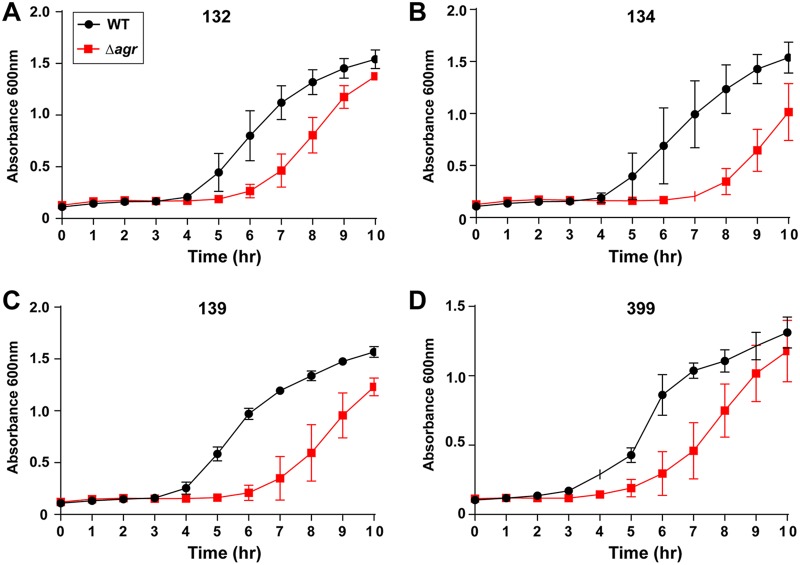
Growth of USA100 strains in the presence of a sub-MIC of vancomycin. WT strains 132 (A), 134 (B), 139 (C), and 399 (D) demonstrated growth superior to that of the paired Δ*agr* mutants when exposed to vancomycin at 2 μg/ml. For at least these strains tested, a functional *agr* system contributes to improved survival in the presence of vancomycin.

The largest differences between the pairs of USA100 strains were observed in the presence of vancomycin. At 2 μg/ml, the upper limit for the MIC (Table 3), the growth of the Δ*agr* mutant lagged behind that of the WT for each of the strain 132, 134, 139, 333, and 399 strain pairs (the results for the strain 132, 134, 139, and 399 strain pairs are shown in Fig. 6A to D, respectively). The two strains in each of the strain 103 and 111 strain pairs did not have any obvious differences in their growth curve patterns, while the two strains in the strain 116 strain pair grew in an inconsistent manner (data not shown). Additional growth curves were performed with clindamycin (32 μg/ml) and daptomycin (20 μg/ml), and in each case, no major differences in growth between the WT and the Δ*agr* mutant were observed.

## DISCUSSION

The *agr* peptide quorum-sensing system is a well-described virulence regulator of CA-MRSA (14). In this study, our goal was to understand the contribution of *agr* to the virulence and antibiotic resistance of clinical HA-MRSA isolates, specifically, those of the USA100 group, which have a more divergent *agr* type II system and which make an AIP-II signal (Fig. 1). Overall, we found that hemolysin and exoenzyme production among the USA100 isolates was dependent on the *agr* system, with some strain-specific exceptions, and the USA100 isolates triggered dermonecrosis in a murine skin infection model in an *agr*-dependent manner. The contribution of *agr* to USA100 antibiotic resistance was limited in terms of MIC levels, but surprisingly, many of the *agr* mutants exhibited less tolerance to vancomycin than their WT counterparts.

The *agr* type II system is the most divergent from the common type I system in terms of sequence and signal structure (Fig. 1B). Despite the variance, the kinetics and behavior of the regulatory system in USA100 isolates shared many similarities to those published for laboratory strains and CA-MRSA. The *agr* activation profile between USA100 and CA-MRSA isolates was similar in timing, although it was weaker in overall output in USA100 isolates (Fig. 2). However, the size of the lesions in a skin infection model correlated well with those that we have observed with USA300 strains (36–38, 40), which could be due to our selection of USA100 isolates with higher alpha-toxin activity (Fig. 1C). Some USA100 *agr* mutants showed extensive background hemolysis, and in the case of strain 399, this could have been due to an intact sequence for *hlb*, encoding β-hemolysin (31). However, these results were not reproducible in the strain 116 strain pair, suggesting that background hemolysis is potentially due to another factor. Similarly, there was a surprising level of background protease activity in many of the USA100 Δ*agr* mutants. The reason for this is not clear, but it could be due to variance in aureolysin (Aur) metalloprotease activity. Aur is one of the primary casein-cleaving enzymes (43) and is under only limited *agr* regulatory control (44). Therefore, the background casein cleavage in Δ*agr* mutants (Fig. 3B) could be due to low levels of Aur production.

Our study also aimed to determine whether or not the *agr* system is involved in USA100 antibiotic resistance. There are previous data to suggest that the *agr* system may have a role in altering antibiotic resistance patterns, as evidenced by the increased survival of *agr* mutants when exposed to antibiotics (18, 23) and also the development of vancomycin-intermediate resistance (vancomycin-intermediate S. aureus) when the *agr* system is dysfunctional (45). Standard MIC testing of the WT strains and the Δ*agr* mutants with 13 different antibiotics found no significant differences in resistance (Table 3), except for resistance to tetracycline, which was used to construct the Δ*agr*::TetM mutation. In a more careful fitness assessment in the presence of low antibiotic levels, the WT and Δ*agr* mutants displayed no growth differences in the presence of clindamycin or daptomycin, which in the case of daptomycin is contrary to the information previously published in the literature (18, 42). When the USA100 isolates were grown in the presence of low levels of vancomycin, some differences in the response were observed. For 5 of the 8 USA100 isolate pairs tested (Fig. 6), the WT grew substantially faster than the Δ*agr* mutants, which seems in contrast to the *agr* dysfunction correlating with vancomycin heteroresistance (45). However, the growth assays with a sub-MIC of vancomycin performed in this study are somewhat different from a heteroresistance assessment. Of the remaining 3 USA100 isolate pairs that showed no significant differences in growth with vancomycin, the WT isolates grew faster than the Δ*agr* mutants in the presence of a sub-MIC of cefoxitin in two of these pairs (Fig. 5). Overall, in the USA100 isolates, our findings suggest that a functional *agr* system is important for optimal survival in the presence of antibiotic stress. We speculate that some of the differences from findings previously reported in the literature are due to strain selection (18, 23, 45). The activity of *agr* across USA100 isolates varies widely (Fig. 1C), and in our study, we focused only on isolates with a robust and functional *agr* system.

As MRSA continues to cause infections worldwide, there is a need to understand both the virulence and resistance mechanisms of the common CA-MRSA strains and the underappreciated impact of HA-MRSA. Among the HA-MSRA strains, strains in the USA100 group are the most common and are known to be highly antibiotic resistant, and they continue to represent a significant burden to the health care system. Our work shows that the virulence properties of the *agr* type II system largely parallel those of the type I system, but the contribution to antibiotic resistance is less clear and in need of further investigation. Previous work has shown that blocking the *agr* system could be a potential target for reducing the severity of infection (36, 38, 40), and this could be applied to the treatment of USA100 infections. With the high level of antibiotic resistance in these strains and the ongoing interest in antibiotic stewardship (46), the development of new approaches to treat multidrug-resistant infections is important.

## MATERIALS AND METHODS

### USA100 strains.

The MRSA strains used in this study had previously been isolated from patients participating in surveillance studies coordinated by the University of Iowa Molecular Epidemiology Laboratory. These strains included both colonizing strains isolated from the nasopharynx and invasive strains, mostly from blood samples.

### *agr* mutant construction.

The Δ*agr*::TetM construct (47) was created by crossing Δ*agr*::TetM by bacteriophage transduction as previously described (48). Successful transduction of the tetracycline resistance cassette was confirmed by DNA PCR. Chromosomal DNA was purified using a Qiagen PureGene kit along with a modified manufacturer protocol. DNA PCR was then performed using Crimson *Taq* DNA polymerase along with RNAIII downstream primer 5′-GTATAAATAAGAAGCGCCCGAAATA-3′ and *agrA* downstream primer 5′-CCAGCTATACAGTGCATTTGCTAGT-3′, with the expected product size for the wild type being 3,823 bp. Deletion of the *agr* operon and insertion of the tetracycline resistance cassette were confirmed after gel electrophoresis, which demonstrated a shorter product in the *agr* knockout strains, as expected.

### Luminescence reporters and assay.

The *agr* P3-*lux* reporter plasmid pAmiAgrP3 (49) was purified from restriction-negative S. aureus strain RN4220 and moved by electroporation into strains LAC (USA300) and MW2 (USA400) and the USA100 strains 116 and 134, using a previously described transformation method (50). Overnight cultures were diluted 1:100 in triplicate into tryptic soy broth (TSB) supplemented with chloramphenicol (10 μg/ml) in a black 96-well plate and grown at 37°C and 1,000 rpm in a Stuart humidified microtiter plate shaker. The optical density at 600 nm (OD_600_) and luminescence were monitored using a Tecan Infinite M Plex plate reader.

### Fluorescence reporters and assay.

The *agr* reporter plasmid pDB59 was transduced from restriction-negative S. aureus strain RN4220 and into strains LAC (USA300) and MW2 (USA400) and the USA100 hospital strains using phage Φ11. Overnight cultures were diluted 1:500 in triplicate into TSB supplemented with chloramphenicol (10 μg/ml) in a black 96-well plate and grown at 37°C and 1,000 rpm in a Stuart humidified microtiter plate shaker. The OD_600_ and the fluorescence at 535 nm following excitation at 492 nm were monitored using a Tecan Infinite M Plex plate reader.

### Alpha-toxin lysis assay.

S. aureus lysis of rabbit red blood cells was performed as previously described (24).

### Hemolysis assay.

To test the hypothesis of *agr* system control of virulence factor expression, hemolysis assays were performed. Each WT strain and *agr* knockout strain pair was plated on sheep blood agar and incubated overnight at 37°C; single colonies were subcultured in TSB and again incubated overnight at 37°C. Each WT and *agr* mutant culture was then diluted 1:100 in TSB and grown to reach an OD_600_ of 3 (approximately 3 to 4 h). Triplicate samples were grown for each strain pair. Subsequently, three samples were plated for each WT and mutant strain on sheep blood agar; each sample contained 2 μl of the culture described above. The samples were allowed to dry and were then incubated for 24 h at 37°C. At 24 h, the diameter of each bacterial colony and the diameter of hemolysis were measured with calipers. These measurements were taken again at 48 h, after the plates were allowed to incubate at 37°C for an additional 24 h. A total of 9 replicates were performed for each WT strain and its paired *agr* mutant strain.

### Protease assay.

Protease assays were completed to further test the hypothesis of *agr* control of virulence factor expression. These experiments were performed with a Pierce fluorescent protease assay kit (Thermo Scientific), which assesses protease activity using fluorescence resonance energy transfer (FRET). For these assays, each WT and mutant strain was cultured overnight in TSB. Each of these cultures was then diluted 1:100 in TSB and grown to an OD_600_ of 1.5. One milliliter of culture at an OD_600_ of 1.5 for each WT and mutant sample was centrifuged at 13,000 × *g* for 1 min. One hundred microliters of the supernatant was transferred to a 96-well plate in triplicate for each wild type and mutant pair; 100 μl of a working reagent containing fluorescent-labeled casein was then added to each well, and the plates were allowed to incubate for 1 h, at which time the plates were analyzed in a fluorescent plate reader (Tecan Infinite 200) at wavelengths of between 485 and 538 nm. Tosylsulfonyl phenylalanyl chloromethyl ketone-treated trypsin (0.5 μg/ml) was used to generate a standard curve, which was then used to calculate protease activity for casein as a trypsin equivalent for each WT and mutant strain. Biological and technical replicates were performed, for a total of 9 data points for each WT and mutant isolate.

### Murine infection model.

Murine models were used to test the virulence of both the WT and *agr* mutant strains as we previously reported (36–38, 40). Cultures were grown overnight at 37°C in a shaking incubator set at 200 rpm from frozen glycerol stocks for two selected wild-type and *agr* mutant strain pairs (strains 116 and 399) in 5 ml of TSB. Subcultures were prepared to a total volume of 350 ml, consisting of 34.65 ml of TSB and 350 μl of an overnight culture, and then grown to an OD_600_ of 0.5 at 37°C in a shaking incubator at 200 rpm. Ten milliliters of this culture was then added to 40 ml of Dulbecco’s phosphate-buffered saline (DPBS) and centrifuged at 3,500 rpm for 10 min. The pelleted cells were then resuspended in sterile saline to reach a concentration of 2 × 10^7^ CFU/50 μl and placed on ice. Five BALB/c mice were used to test each strain, resulting in the use of 20 mice in total. Mice were anesthetized with 1 to 5% isoflurane. The abdominal skin of each mouse was shaven with a microtome blade; the mice were then labeled, weighed, and prepped for sterile injection. Insulin syringes were used to intradermally inoculate each mouse with 50 μl of a specified S. aureus suspension. The mice were monitored daily, with the lesion size (in square centimeters) as well as weight loss being recorded as markers of disease for up to 2 weeks. All mouse experiments were approved by the University of Iowa Institutional Animal Care and Use Committee.

### Determination of MIC and antibiotic resistance patterns.

Comparison of antibiotic resistance patterns was performed using Etest strips (bioMérieux USA) to determine the MICs of cefoxitin, clindamycin, daptomycin, erythromycin, gentamicin, levofloxacin, linezolid, oxacillin, quinupristin-dalfopristin, rifampin, tetracycline, trimethoprim-sulfamethoxazole, and vancomycin. Triplicate testing for each isolate (the wild-type and null mutant isolates) was performed per the manufacturer’s protocol, and a methicillin-sensitive Staphylococcus aureus subsp. *aureus* Rosenbach (ATTC 29213) strain served as a quality control for these experiments.

To examine more subtle differences between these isolates in the presence of selected antibiotics, growth curves were performed for all strain pairs. The antibiotics selected for these studies were cefoxitin, clindamycin, daptomycin, and vancomycin. The WT and *agr* mutant strains were cultured overnight in TSB at 37°C. Ninety-six-well plates were prepared with serially diluted concentrations of antibiotic added to TSB. For each antibiotic, seven different concentrations were tested; in addition, one control sample without antibiotic was used for each strain. The concentrations tested for cefoxitin were 1 mg/ml, 500 μg/ml, 250 μg/ml, 125 μg/ml, 62.5 μg/ml, 31.25 μg/ml, and 15.625 μg/ml; those tested for clindamycin were 16 μg/ml, 8 μg/ml, 4 μg/ml, 2 μg/ml, 1 μg/ml, 0.5 μg/ml, and 0.25 μg/ml; those tested for daptomycin were 100 μg/ml, 50 μg/ml, 20 μg/ml, 16 μg/ml, 8 μg/ml, 4 μg/ml, and 2 μg/ml; and those tested for vancomycin were 2 μg/ml, 1 μg/ml, 0.5 μg/ml, 0.25 μg/ml, 0.125 μg/ml, and 0.625 μg/ml. Bacteria were grown from a 1:50 dilution in each well containing the respective concentration of antibiotic. These plates were then incubated at 37°C and shaken at 1,000 rpm. The absorbance (OD_600_) was measured by use of a microtiter plate reader (Tecan Infinite 200) starting at hour 0 and then hourly thereafter until the bacteria had reached stationary phase (8 to 10 h); measurements were then used to generate growth curves for each strain at the various antibiotic concentrations.

### Statistical analysis.

GraphPad Prism (version 7) software was used to perform statistical analysis of the hemolysis, protease, and animal data. Student's *t* test was chosen to calculate the difference in hemolysis and proteolysis between the wild-type strain and paired *agr* mutant as well as the murine data comparing lesion size and weight loss between the groups challenged with the WT and the *agr* mutant. A *P* value of <0.05 was chosen to designate a statistically significant difference between values. Throughout the work, error bars represent standard deviations.
